# Supplementary material for: The interactome of intact mitochondria by cross-linking mass spectrometry provides evidence for coexisting respiratory supercomplexes
Source: Mol Cell Proteomics. 2017 Dec 8;17(2):216–32. doi: 10.1074/mcp.RA117.000470 (PMC5795388; doi:10.1074/mcp.RA117.000470)
Supplement: Supplemental Data [file supp_RA117.000470_133922_0_supp_23978_fzffwf.zip › spectra_annotation/mito_spectra_annotation/94-1-3-1-5-1.pdf]

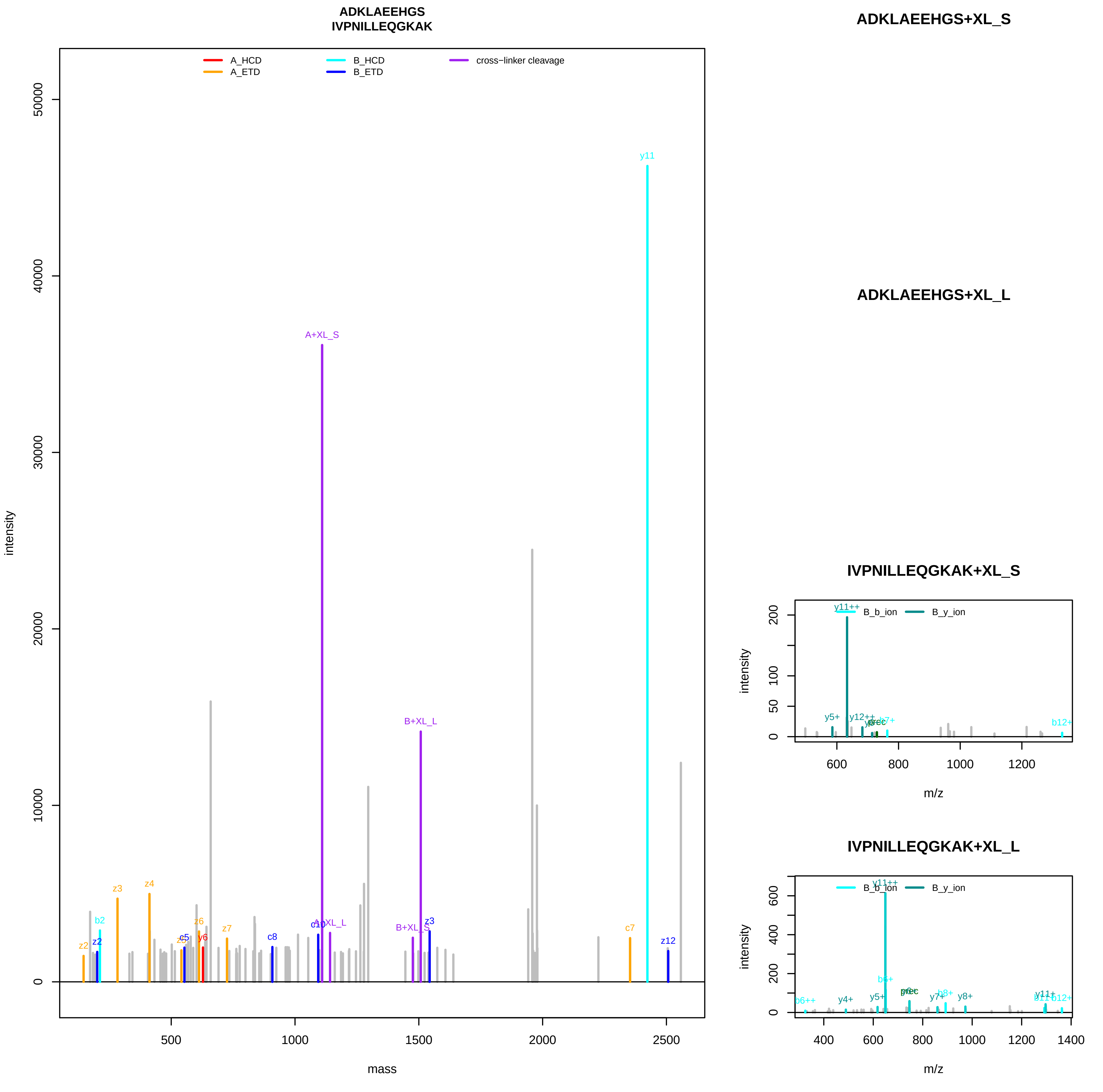

ADKLAEEHGS
IVPNILLEQGKAK
A_HCD
A_ETD
B_HCD
B_ETD
cross−linker cleavage
intensity
mass
0
10000
20000
30000
40000
50000
500
1000
1500
2000
2500
y11
A+XL_S
B+XL_L
z2
b2
z2
z3
z4
c5
z6
y6
z7
c8
c10
A+XL_L
B+XL_S
z3
c7
z12
ADKLAEEHGS+XL_S
ADKLAEEHGS+XL_L
IVPNILLEQGKAK+XL_S
B_b_ion
B_y_ion
y11++
y5+
y12++
prec
b7+
b12+
0
50
100
200
600
800
1000
1200
m/z
IVPNILLEQGKAK+XL_L
B_b_ion
B_y_ion
y11++
b6+
b6++
y4+
y5+
prec
y7+
b8+
y8+
y11+
b12+
0
200
400
600
400
600
800
1000
1200
1400
m/z
